# Supplementary material for: Embedded timing and alert device triggered by total dissolved solids (TDS) for monitoring disinfection duration in acidic electrolyzed oxidizing water
Source: PLoS One. 2026 May 18;21(5):e0349390. doi: 10.1371/journal.pone.0349390 (PMC13183236; doi:10.1371/journal.pone.0349390)
Supplement: S2 File — (DOCX) [file pone.0349390.s002.docx]

#include <WiFi.h>

#include <WebServer.h>

#include <SPIFFS.h>

#include <time.h>

#include <DNSServer.h>

#include <Preferences.h>

// \-\-- Hardware Pin Definitions \-\--

#define SENSOR_PIN 14 // Sensor input pin, triggered by high level

#define BUTTON_PIN 4 // Button pin, internal pull-up, low when pressed (RTC IO pin)

#define LED_PIN 17 // LED indicator pin

// \-\-- WiFi and AP Configuration \-\--

const char* apSSID = "ESP32-Config";

const char* apPassword = "12345678";

// \-\-- File and Storage Configuration \-\--

Preferences preferences;

const char* filename = "/sensor_log.txt";

const char* initFlagFile = "/initialized.flag";

const int MAX_LOG_ENTRIES = 200; // Maximum number of log entries

// \-\-- Server and Network Objects \-\--

WebServer server(80);

DNSServer dnsServer;

// \-\-- Global State Variables \-\--

bool isConnected = false;

bool configMode = false;

bool dataViewMode = false;

bool sensorTimingMode = false; // Sensor timing phase (default double flash)

bool sensorHighOver2min = false; // Flag: whether sensor high level exceeds 2 minutes (used for LED always on)

unsigned long dataViewStartTime = 0;

const unsigned long DATA_VIEW_TIMEOUT = 5 * 60 * 1000; // Data view timeout (5 minutes)

const unsigned long SENSOR_HIGH_STAGE1 = 2 * 60 * 1000; // Stage 1 duration (2 minutes, double flash)

// LED Control Variables

unsigned long ledPrevMillis = 0;

bool ledState = LOW;

const unsigned long LED_DBL_FLASH_ON = 500; // Double flash single ON duration

const unsigned long LED_DBL_FLASH_OFF = 1000; // Interval between double flash groups

const unsigned long LED_SLOW_PERIOD = 2000; // Slow flash period for config/data mode

int dblFlashCount = 0;

String wifiSSID = "";

String wifiPassword = "";

String endTimeStr = "NTP Time Unavailable"; // Sensor high level end time (for logging)

// \-\-- Function Declarations \-\--

void connectToWiFi();

void enterConfigMode();

void enterDataViewMode();

bool saveWifiConfig();

bool loadWifiConfig();

void goToDeepSleep();

void handleRoot();

void handleDownload();

void handleData();

void handleClearLog();

void handleWifiConfig();

void handleSaveWifiConfig();

void handleReboot();

void handleNotFound();

void initializeLogFile();

void limitLogFileSize();

void logSensorTrigger(String endTimeStr, unsigned long totalDurationSec);

void setupHardware();

void checkButtonPress();

void ledBlink(int times, int period);

void startWebServerForDataView();

void updateLED();

// Helper class: String splitting

class StringArray {

private:

String* data;

int capacity;

int m_size;

public:

StringArray() : data(nullptr), capacity(0), m_size(0) {}

~StringArray() { delete[] data; }

void add(const String& s);

String get(int index) const;

int size() const { return m_size; }

};

void StringArray::add(const String& s) {

if (m_size >= capacity) {

int newCapacity = capacity == 0 ? 4 : capacity * 2;

String* newData = new String[newCapacity];

for (int i = 0; i < m_size; i++) newData[i] = data[i];

delete[] data;

data = newData;

capacity = newCapacity;

}

data[m_size++] = s;

}

String StringArray::get(int index) const {

if (index >= 0 && index < m_size) return data[index];

return "";

}

StringArray splitString(const String& str, const String& delimiter);

StringArray splitString(const String& str, const String& delimiter) {

StringArray result;

int startIndex = 0;

int endIndex = str.indexOf(delimiter);

while (endIndex != -1) {

String token = str.substring(startIndex, endIndex);

if (token.length() > 0) result.add(token);

startIndex = endIndex + delimiter.length();

endIndex = str.indexOf(delimiter, startIndex);

}

String lastToken = str.substring(startIndex);

if (lastToken.length() > 0) result.add(lastToken);

return result;

}

void setup() {

Serial.begin(115200);

Serial.println("\nESP32 Sensor Logger waking up...");

setupHardware();

esp_sleep_wakeup_cause_t wakeup_reason = esp_sleep_get_wakeup_cause();

// Initialize file system and configuration

if(!SPIFFS.begin(true)) {

Serial.println("An Error has occurred while mounting SPIFFS");

}

preferences.begin("wifi-config", false);

loadWifiConfig();

initializeLogFile();

switch (wakeup_reason) {

case ESP_SLEEP_WAKEUP_EXT0: { // Woken up by sensor trigger (high level)

Serial.println("Wakeup caused by sensor trigger. Starting timing...");

connectToWiFi(); // Connect to WiFi in advance to get NTP time quickly later

// 1. Record the millisecond timestamp when sensor goes high

unsigned long sensorHighStartMillis = millis();

// 2. Enter sensor timing mode (default double flash)

sensorTimingMode = true;

ledPrevMillis = millis();

dblFlashCount = 0;

// 3. Continuously monitor sensor: wait until pin goes low, control LED in stages

while (digitalRead(SENSOR_PIN) == HIGH) {

// If exceeds 2 minutes: switch to LED always on

if (!sensorHighOver2min && (millis() - sensorHighStartMillis >= SENSOR_HIGH_STAGE1)) {

sensorHighOver2min = true;

Serial.println("Sensor high over 2 minutes. LED switch to ON.");

}

updateLED(); // Update LED state based on current stage (double flash / always on)

delay(100); // Reduce check frequency to save resources

}

// 4. Calculate total high level duration (milliseconds to seconds)

unsigned long totalHighDurationSec = (millis() - sensorHighStartMillis) / 1000;

Serial.printf("Total sensor high duration: %lu seconds\n", totalHighDurationSec);

// 5. At sensor end, get NTP time as "end time point"

if (isConnected) {

struct tm timeinfo;

if(getLocalTime(&timeinfo)){

char buffer[20];

strftime(buffer, sizeof(buffer), "%Y-%m-%d %H:%M:%S", &timeinfo);

endTimeStr = String(buffer);

}

}

Serial.printf("Sensor high end time: %s\n", endTimeStr.c_str());

// 6. Log to file: end time point + total duration

logSensorTrigger(endTimeStr, totalHighDurationSec);

// 7. LED stays on for 5 seconds to indicate "logging complete", then turns off

digitalWrite(LED_PIN, HIGH);

delay(5000);

digitalWrite(LED_PIN, LOW);

// 8. Enter deep sleep

goToDeepSleep();

break;

}

case ESP_SLEEP_WAKEUP_EXT1: { // Woken up by button press

Serial.println("Wakeup caused by button press.");

checkButtonPress();

break;

}

case ESP_SLEEP_WAKEUP_TIMER: { // Woken up by hardware timer (backup)

Serial.println("Wakeup caused by hardware timer.");

goToDeepSleep();

break;

}

default: { // First boot or other reasons

Serial.println("First boot or unknown wakeup reason. Entering deep sleep.");

goToDeepSleep();

break;

}

}

}

void loop() {

if (configMode || dataViewMode) {

if(configMode) dnsServer.processNextRequest();

server.handleClient();

updateLED(); // Slow flash for config/data mode

// Sleep after data view timeout

if (dataViewMode && (millis() - dataViewStartTime >= DATA_VIEW_TIMEOUT)) {

Serial.println("Data view mode timed out. Going to sleep...");

goToDeepSleep();

}

}

}

// \-\-- WiFi and Mode Management Functions \-\--

void connectToWiFi() {

if (wifiSSID == "") {

Serial.println("No WiFi credentials available.");

isConnected = false;

return;

}

Serial.println("Connecting to WiFi: " + wifiSSID);

WiFi.mode(WIFI_STA);

WiFi.begin(wifiSSID.c_str(), wifiPassword.c_str());

int attempts = 0;

while (WiFi.status() != WL_CONNECTED && attempts < 20) {

delay(500);

Serial.print(".");

attempts++;

}

if (WiFi.status() == WL_CONNECTED) {

isConnected = true;

Serial.println("\nConnected! IP: " + WiFi.localIP().toString());

configTime(8 * 3600, 0, "pool.ntp.org", "time.windows.com");

} else {

isConnected = false;

Serial.println("\nFailed to connect to WiFi.");

}

}

void enterConfigMode() {

Serial.println("Entering configuration mode...");

configMode = true;

dataViewMode = false;

sensorTimingMode = false;

sensorHighOver2min = false;

ledPrevMillis = millis();

ledState = LOW;

digitalWrite(LED_PIN, ledState);

WiFi.mode(WIFI_AP);

WiFi.softAP(apSSID, apPassword);

IPAddress apIP = WiFi.softAPIP();

Serial.print("AP IP: ");

Serial.println(apIP);

dnsServer.start(53, "*", apIP);

server.on("/", handleRoot);

server.on("/wifi-config", HTTP_GET, handleWifiConfig);

server.on("/save-wifi-config", HTTP_POST, handleSaveWifiConfig);

server.onNotFound(handleNotFound);

server.begin();

Serial.println("Config mode web server started.");

}

void enterDataViewMode() {

Serial.println("Entering data view mode...");

dataViewMode = true;

configMode = false;

sensorTimingMode = false;

sensorHighOver2min = false;

ledPrevMillis = millis();

ledState = LOW;

digitalWrite(LED_PIN, ledState);

if (wifiSSID != "") {

WiFi.mode(WIFI_STA);

WiFi.begin(wifiSSID.c_str(), wifiPassword.c_str());

delay(5000);

WiFi.disconnect(true);

delay(1000);

}

WiFi.mode(WIFI_AP);

WiFi.softAP(apSSID, apPassword);

IPAddress apIP = WiFi.softAPIP();

Serial.print("Data View AP IP: ");

Serial.println(apIP);

dataViewStartTime = millis();

Serial.println("Data view mode will sleep after 5 minutes.");

startWebServerForDataView();

}

bool saveWifiConfig() {

preferences.putString("ssid", wifiSSID);

preferences.putString("password", wifiPassword);

return true;

}

bool loadWifiConfig() {

wifiSSID = preferences.getString("ssid", "");

wifiPassword = preferences.getString("password", "");

return wifiSSID != "";

}

void goToDeepSleep() {

Serial.println("Going to deep sleep...");

digitalWrite(LED_PIN, LOW); // Force LED off before sleep

server.stop();

dnsServer.stop();

WiFi.disconnect(true);

WiFi.mode(WIFI_OFF);

preferences.end();

SPIFFS.end();

// Configure wake-up sources

esp_sleep_enable_ext0_wakeup(GPIO_NUM_14, 1); // Wake on sensor high level

const uint64_t button_mask = (1ULL << BUTTON_PIN);

esp_sleep_enable_ext1_wakeup(button_mask, ESP_EXT1_WAKEUP_ALL_LOW); // Wake on button low level

esp_deep_sleep_start();

}

// \-\-- Web Server Handlers \-\--

void handleRoot() {

if(configMode) {

server.sendHeader("Location", "/wifi-config");

server.send(302, "text/plain", "Redirecting to config");

return;

}

if (SPIFFS.exists("/index.html")) {

File file = SPIFFS.open("/index.html");

server.streamFile(file, "text/html");

file.close();

} else {

server.send(200, "text/html",

"<html><body>"

"<h1>ESP32 Sensor Logger</h1>"

"<p>Index.html not found.</p>"

"<p><a href='/data'>View Log</a> | <a href='/download'>Download Log</a></p>"

"</body></html>");

}

}

void handleWifiConfig() {

if (SPIFFS.exists("/wifi_config.html")) {

File file = SPIFFS.open("/wifi_config.html");

server.streamFile(file, "text/html");

file.close();

} else {

Serial.println("Warning: wifi_config.html not found, serving fallback page.");

String html =

"<html><head><title>WiFi Config</title></head><body>"

"<h1>WiFi Configuration</h1>"

"<form action='/save-wifi-config' method='POST'>"

"<label>SSID: <input type='text' name='ssid' value='" + wifiSSID + "' required></label><br>"

"<label>Password: <input type='password' name='password' value='" + wifiPassword + "'></label><br>"

"<button type='submit'>Save</button>"

"</form>"

"</body></html>";

server.send(200, "text/html", html);

}

}

void handleSaveWifiConfig() {

wifiSSID = server.arg("ssid");

wifiPassword = server.arg("password");

if(saveWifiConfig()) {

server.send(200, "text/html",

"<html><body>"

"<h1>WiFi Saved</h1>"

"<p>Rebooting in 5 seconds...</p>"

"<script>setTimeout(function(){ window.location.href = '/'; }, 5000);</script>"

"</body></html>");

delay(3000);

ESP.restart();

} else {

server.send(500, "text/plain", "Failed to save.");

}

}

void handleDownload() {

if (SPIFFS.exists(filename)) {

File file = SPIFFS.open(filename, FILE_READ);

server.sendHeader("Content-Disposition", "attachment; filename=sensor_log.txt");

server.streamFile(file, "text/plain");

file.close();

} else {

server.send(404, "text/plain", "Log file not found.");

}

}

void handleData() {

if (SPIFFS.exists(filename)) {

File file = SPIFFS.open(filename, FILE_READ);

server.streamFile(file, "text/plain");

file.close();

} else {

server.send(404, "text/plain", "Log file not found.");

}

}

void handleClearLog() {

File file = SPIFFS.open(filename, FILE_WRITE);

if(file) {

file.println("End Time, Total High Duration (seconds)"); // Log header: end time + total duration

file.close();

server.send(200, "text/plain", "Log cleared.");

} else {

server.send(500, "text/plain", "Failed to clear log.");

}

}

void handleReboot() {

server.send(200, "text/html", "<html><body><h1>Rebooting...</h1></body></html>");

delay(1000);

ESP.restart();

}

void handleNotFound() {

server.send(404, "text/plain", "Not Found");

}

// \-\-- File and Log Handling Functions \-\--

void initializeLogFile() {

if (SPIFFS.exists(initFlagFile)) return;

// Initialize log header: end time point + total high level duration in seconds

File file = SPIFFS.open(filename, FILE_WRITE);

if (file) {

file.println("End Time, Total High Duration (seconds)");

file.close();

}

// Create initialization flag file

File flagFile = SPIFFS.open(initFlagFile, FILE_WRITE);

if (flagFile) {

flagFile.println("Initialized");

flagFile.close();

}

}

void limitLogFileSize() {

if (!SPIFFS.exists(filename)) return;

File file = SPIFFS.open(filename, FILE_READ);

if (!file) return;

String header = file.readStringUntil('\n'); // Keep header

String allLines = "";

int lineCount = 0;

while (file.available()) {

String line = file.readStringUntil('\n');

if (line.length() > 0) {

allLines += line + "\n";

lineCount++;

}

}

file.close();

// If exceeds max entries, keep the latest MAX_LOG_ENTRIES lines

if (lineCount > MAX_LOG_ENTRIES - 1) {

Serial.println("Log file too big, trimming...");

StringArray lines = splitString(allLines, "\n");

int linesToKeep = MAX_LOG_ENTRIES - 1;

int startIndex = lines.size() - linesToKeep;

String newContent = header + "\n";

for (int i = startIndex; i < lines.size(); i++) {

if (lines.get(i).length() > 0) newContent += lines.get(i) + "\n";

}

File writeFile = SPIFFS.open(filename, FILE_WRITE);

if (writeFile) {

writeFile.print(newContent);

writeFile.close();

}

}

}

// Core function: log "end time point" and "total high level duration in seconds" to file

void logSensorTrigger(String endTimeStr, unsigned long totalDurationSec) {

limitLogFileSize(); // Trim log size first to avoid overflow

File file = SPIFFS.open(filename, FILE_APPEND);

if(file){

// Format: end time, total duration in seconds (e.g., 2024-10-01 14:35:00, 300)

file.printf("%s, %lu\n", endTimeStr.c_str(), totalDurationSec);

file.close();

Serial.printf("Logged: %s | Total Duration=%lu seconds\n", endTimeStr.c_str(), totalDurationSec);

}

}

// \-\-- Hardware and Low Power Functions \-\--

void setupHardware() {

pinMode(SENSOR_PIN, INPUT);

pinMode(BUTTON_PIN, INPUT_PULLUP);

pinMode(LED_PIN, OUTPUT);

digitalWrite(LED_PIN, LOW);

}

void checkButtonPress() {

delay(50); // Debounce

if (digitalRead(BUTTON_PIN) == HIGH) {

goToDeepSleep();

}

Serial.println("Button pressed, checking duration...");

unsigned long pressStartTime = millis();

while (digitalRead(BUTTON_PIN) == LOW && millis() - pressStartTime < 5000) {

delay(50);

}

unsigned long pressDuration = millis() - pressStartTime;

if (pressDuration < 5000) { // Short press: blink 3 times

Serial.println("Short press detected.");

ledBlink(3, 1000);

goToDeepSleep();

} else { // Long press: enter config or data mode

Serial.println("Long press detected.");

connectToWiFi();

if (!isConnected) {

Serial.println("Cannot connect to WiFi, entering config mode.");

enterConfigMode();

} else {

Serial.println("WiFi connected briefly, now entering data view mode.");

enterDataViewMode();

}

}

}

// Short press only: blink LED n times (blocking)

void ledBlink(int times, int period) {

for (int i = 0; i < times; i++) {

digitalWrite(LED_PIN, HIGH);

delay(period / 2);

digitalWrite(LED_PIN, LOW);

delay(period / 2);

}

}

void startWebServerForDataView() {

server.on("/", handleRoot);

server.on("/data", handleData);

server.on("/download", handleDownload);

server.on("/clear", HTTP_GET, handleClearLog);

server.on("/reboot", HTTP_GET, handleReboot);

server.on("/wifi-config", HTTP_GET, handleWifiConfig);

server.on("/save-wifi-config", HTTP_POST, handleSaveWifiConfig);

server.onNotFound(handleNotFound);

server.begin();

Serial.println("Data view mode web server started.");

}

// Core update: LED state control (staged)

void updateLED() {

unsigned long currentMillis = millis();

// 1. Highest priority: sensor high level exceeds 2 minutes → LED always on

if (sensorHighOver2min) {

digitalWrite(LED_PIN, HIGH);

return;

}

// 2. Sensor stage 1 (0-2 minutes) → LED double flash

if (sensorTimingMode) {

switch (dblFlashCount) {

case 0: // Interval between double flash groups (off)

if (currentMillis - ledPrevMillis >= LED_DBL_FLASH_OFF) {

ledState = HIGH;

digitalWrite(LED_PIN, ledState);

ledPrevMillis = currentMillis;

dblFlashCount = 1;

}

break;

case 1: // First flash on

if (currentMillis - ledPrevMillis >= LED_DBL_FLASH_ON) {

ledState = LOW;

digitalWrite(LED_PIN, ledState);

ledPrevMillis = currentMillis;

dblFlashCount = 2;

}

break;

case 2: // First flash off (interval between flashes)

if (currentMillis - ledPrevMillis >= LED_DBL_FLASH_ON) {

ledState = HIGH;

digitalWrite(LED_PIN, ledState);

ledPrevMillis = currentMillis;

dblFlashCount = 3;

}

break;

case 3: // Second flash on

if (currentMillis - ledPrevMillis >= LED_DBL_FLASH_ON) {

ledState = LOW;

digitalWrite(LED_PIN, ledState);

ledPrevMillis = currentMillis;

dblFlashCount = 0;

}

break;

}

}

// 3. Config/data mode → LED slow flash (2-second period)

else if (configMode || dataViewMode) {

if (currentMillis - ledPrevMillis >= LED_SLOW_PERIOD / 2) {

ledState = !ledState;

digitalWrite(LED_PIN, ledState);

ledPrevMillis = currentMillis;

}

}

// 4. Other modes → LED off

else {

if (ledState != LOW) {

ledState = LOW;

digitalWrite(LED_PIN, ledState);

}

}

}
